# Delayed Goblet Cell Hyperplasia, Acetylcholine Receptor Expression, and Worm Expulsion in SMC-Specific IL-4Rα–Deficient Mice

**DOI:** 10.1371/journal.ppat.0030001

**Published:** 2007-01-12

**Authors:** William G. C Horsnell, Antony J Cutler, Claire J Hoving, Helen Mearns, Elmarie Myburgh, Berenice Arendse, Fred D Finkelman, Gary K Owens, Dave Erle, Frank Brombacher

**Affiliations:** 1 Division of Immunology, Institute of Infectious Disease and Molecular Medicine, Faculty of Health Sciences, University of Cape Town, Cape Town, South Africa; 2 Department of Medicine, University of Cincinnati College of Medicine, Cincinnati, Ohio, United States of America; 3 Department of Molecular Physiology and Biological Physics, University of Virginia Health Sciences Center, Charlottesville, Virginia, United States of America; 4 Lung Biology Center, Department of Medicine, University of California San Francisco, San Francisco, California, United States of America; National Institutes of Health, United States of America

## Abstract

Interleukin 4 receptor α (IL-4Rα) is essential for effective clearance of gastrointestinal nematode infections. Smooth muscle cells are considered to play a role in the type 2 immune response–driven expulsion of gastrointestinal nematodes. Previous studies have shown in vitro that signal transducer and activator of transcription 6 signaling in response to parasitic nematode infection significantly increases smooth muscle cell contractility. Inhibition of the IL-4Rα pathway inhibits this response. How this response manifests itself in vivo is unknown. In this study, smooth muscle cell IL-4Rα–deficient mice (SM-MHC^Cre^IL-4Rα^−/lox^) were generated and characterized to uncover any role for IL-4/IL-13 in this non–immune cell type in response to Nippostrongylus brasiliensis infection. IL-4Rα was absent from α-actin–positive smooth muscle cells, while other cell types showed normal IL-4Rα expression, thus demonstrating efficient cell-type–specific deletion of the IL-4Rα gene. N. brasiliensis–infected SM-MHC^Cre^IL-4Rα^−/lox^ mice showed delayed ability to resolve infection with significantly prolonged fecal egg recovery and delayed worm expulsion. The delayed expulsion was related to a delayed intestinal goblet cell hyperplasia, reduced T helper 2 cytokine production in the mesenteric lymph node, and reduced M3 muscarinic receptor expression during infection. Together, these results demonstrate that in vivo IL-4Rα–responsive smooth muscle cells are beneficial for N. brasiliensis expulsion by coordinating T helper 2 cytokine responses, goblet hyperplasia, and acetylcholine responsiveness, which drive smooth muscle cell contractions.

## Author Summary

Intestinal parasitic worm infections are a major public health concern, with more than 1 billion people infected worldwide. Symptoms associated with these infections are similar to that of other intestinal illnesses, including irritable bowel syndrome. It is likely that the immune response required to expel the worm can also, when activated inappropriately, cause the symptoms of irritable bowel syndrome. This makes understanding parasitic worm infections important in their own right and also as a model for other intestinal illnesses. In previous studies, we demonstrated the crucial importance of interleukin 4 receptor α (IL-4Rα) responsiveness for worm expulsion in global IL-4Rα–deficient mice. In this study, we specifically addressed the role of IL-4Rα responsiveness in a novel smooth muscle cell–specific IL-4Rα–deficient mouse model. These mice showed decreased ability to control the worm infection, with delayed expulsion and reduced protective immune responses. These data provide compelling evidence for smooth muscle cell IL-4Rα being an important coordinator of both the immune and physiological responses to intestinal worm infections. A proposed model is suggested with IL-4Rα responsiveness on smooth muscle cells coordinating T helper 2 cytokine responses, goblet hyperplasia, and acetylcholine-driven smooth muscle contractions for optimal worm expulsion.

## Introduction

The interleukin (IL)-13/IL-4 receptor α (IL-4Rα)/signal transducer and activator of transcription 6 (STAT-6) signaling pathway is essential in the control of a number of infectious diseases as well as being a key factor in the induction of allergic responses. Signaling through this pathway can either confer protective immunity or mediate tissue damage depending on the antigenic stimuli and the cell-specific response [1]. Previously, our laboratory provided the first description of the effect of a cell-specific deletion of IL-4Rα from macrophages and neutrophils on the host's ability to respond to two parasitic infections [2]. It was demonstrated that such a deletion failed to affect resolution of infection by the nematode *Nippostrongylus brasiliensis,* while mice demonstrated an increased susceptibility to infection by the trematode Schistosoma mansoni. The work presented here describes the effect of a smooth muscle–specific disruption of IL-4Rα expression on the immune response to N. brasiliensis.

Murine infection with N. brasiliensis induces a strong protective host T helper 2 (T_H_2) response for which IL-13 production and signaling through IL-4Rα are essential for successful clearance of infection [3,4]. Infective third-stage N. brasiliensis larva penetrate the skin and migrate via the blood system, to the lungs. Larva emerge from blood vessels and enter the airways, from which they are coughed up and swallowed. Upon reaching the intestine, larva develop into egg-producing adult worms that attach to the small intestine epithelium. BALB/c mice clear N. brasiliensis infection after approximately 9 d [5].

Although essential for expulsion of N. brasiliensis from the intestine, the precise role of IL-4Rα in coordinating the immune and physiological response remains unclear [6]. IL-13/IL-4Rα/STAT-6 signaling is required for the host to produce an effective goblet cell hyperplasia [7]. Disruption of this response impairs the host ability to resolve an N. brasiliensis infection. Additionally, acetylcholine-driven contractions of longitudinal smooth muscle in the intestine are also implicated in playing a role in worm expulsion [8]. A number of in vitro studies have shown that intestinal segments and intestinal smooth muscle cells previously exposed to infection by murine nematode models have increased contractile ability. This contractile ability of intestinal segments and/or smooth muscle cells is abrogated in STAT-6^−/−^ mice. Therefore, the IL-13/IL-4Rα/STAT-6 pathway is necessary for elevated smooth muscle cell contractility required to aid worm expulsion [6,9,10]. Additionally, IL-13/IL-4Rα/STAT-6–dependent smooth muscle cell signaling can induce responses in surrounding tissues [11], as well as inducing smooth muscle cell release of chemokines, such as thymus- and activation-regulated chemokine [12], in order to coordinate early host responses to pathogens. From these studies, it is apparent that both goblet cell and smooth muscle cell responses to nematode infections are coordinated by the host immune response to infection and that this coordination is essential for optimal disease resolution [13].

To date, no studies have been able to demonstrate in vivo the effect of a cell-specific inhibition of the IL-13/IL-4Rα/STAT-6 pathway in smooth muscle cells. Using smooth muscle myosin heavy chain (SM-MHC)^Cre^IL-4Rα^−/lox^ mice, we demonstrate that disrupted IL-4Rα expression in smooth muscle cells influences host immunity to an intestinal nematode infection. The absence of smooth muscle IL-4Rα delays worm expulsion and goblet cell hyperplasia. Furthermore, induction of T_H_2 cytokines is delayed and/or reduced, as is intestinal expression of the M3 acetylcholine receptor, in response to infection with N. brasiliensis.

## Results

**Figure 1:**
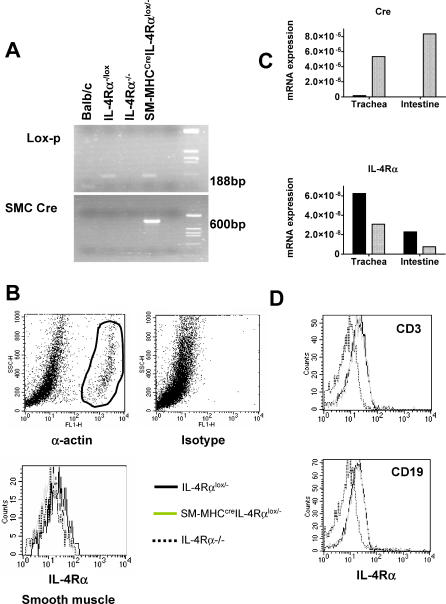
IL-4Rα Expression Is Impaired on Smooth Muscle Cells in SM-MHC^Cre^IL-4Rα^−/lox^ Mice (A) Genomic integrity of the SM-MHC^Cre^IL-4Rα^−/lox^ hemizygous mice was established by PCR. (B) Smooth muscle cells were identified by intracellular α-actin (i) staining versus isotype control (ii). IL-4Rα surface expression was analyzed on gated α-actin–positive cells (iii). (C) cDNA levels in trachea and small intestine of IL-4Rα^−/lox^ (black bars) and SM-MHC^Cre^IL-4Rα^−/lox^ (hatched bars). Data are derived from pooled tissue samples from three mice and are representative of two experiments. (D) IL-4Rα expression on lymphocyte subpopulations of T cells and B cells is unaffected in SM-MHC^Cre^IL-4Rα^−/lox^ mice.

Transgenic mice, expressing Cre recombinase under the control of the smooth muscle cell–specific myosin heavy chain promoter (SM-MHC^Cre^), were backcrossed to the BALB/c genetic background for nine generations and then intercrossed with IL-4Rα^−/−^ and “floxed” IL-4Rα^lox/lox^ BALB/c mice to establish smooth muscle cell–specific IL-4Rα–deficient BALB/c mice (SM-MHC^Cre^IL-4Rα^−/lox^), with one deleted and one floxed IL-4Rα allele (SM-MHC^Cre^IL-4Rα^−/lox^) to increase the efficiency of Cre-mediated site-specific recombination. Mutant mouse strains were identified by PCR genotyping (Figure 1A), and cell specificity of disrupted IL-4Rα expression was confirmed by fluorescence-activated cell sorting analysis (FACS).

IL-4Rα expression was analyzed on α-actin–positive cells derived from aortic cells (Figure 1B). Surface expression of IL-4Rα on α-actin–positive cells was equivalent in SM-MHC^Cre^IL-4Rα^−/lox^ (geometric mean fluorescence [GMF]: 11.02) and global IL-4Rα^−/−^ (GMF: 11.2) mice (Figure 1B). Low levels of expression were present in IL-4Rα^−/lox^ mice (GMF: 18.37). IL-4Rα expression on α-actin–positive smooth muscle cells isolated from small intestine and lung was too low to detect using FACS analysis (unpublished data). However, Cre mRNA was highly expressed in tracheal and intestinal tissue in the SM-MHC^Cre^IL-4Rα^−/lox^ mice. As expected, IL-4Rα^−/lox^ mice demonstrated no Cre expression. In agreement with the smooth muscle specificity of the deletion, IL-4Rα mRNA expression was substantially depressed in both tracheal and intestinal tissue in SM-MHC^Cre^IL-4Rα^−/lox^ mice compared to IL-4Rα^−/lox^ mice (Figure 1C). Importantly, IL-4Rα expression was maintained on CD3^+^ T cells, CD19^+^ B cells (Figure 1D), and macrophages (unpublished data) in smooth muscle cell–specific IL-4Rα knockout mice and equivalent to levels expressed on transgenic Cre-negative IL-4Rα^−/lox^ control littermates. Functional analysis confirmed IL-4Rα responsiveness in these cell types (unpublished data). Together, these results provide convincing support for the specificity of smooth muscle cell disruption of IL-4Rα in SM-MHC^Cre^IL-4Rα^−/lox^ mice, in agreement with previously published data on the characterization of SM-MHC^Cre^ transgenic mice [14].

**Figure 2:**
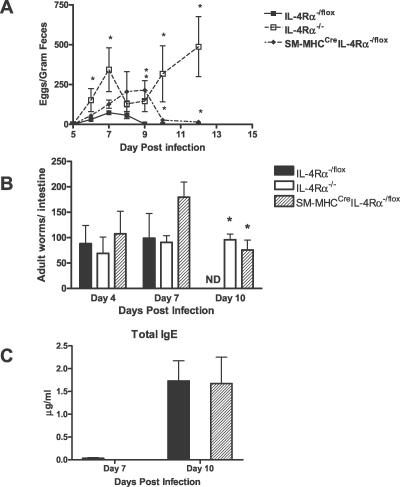
SM-MHC^Cre^IL-4Rα^−/lox^ Mice Have a Delayed Adult Worm Expulsion from the Intestine (A) N. brasiliensis egg production in infected mice was assessed daily from day 5 PI using the modified McMaster technique. (B) Worm burden was established on days 4, 7, and 10 PIn by counting worms in intestines removed from infected mice. (C) Serum IgE antibody responses in IL-4Rα^−/lox^ and SM-MHC^Cre^IL-4Rα^−/lox^ mice are equivalent. Serum from infected mice was taken on days 7 and 10 PI and analyzed for antibody production by ELISA as described in Materials and Methods. *Significant differences from IL-4Rα^−/lox^ mice (*p* < 0.05); data are representative of four separate experiments.

To investigate a possible role of IL-4/IL-13–stimulated smooth muscle cells in nematode infections, comparative infection studies with the gastrointestinal nematode N. brasiliensis were performed. Worm fecundity in the host was followed by determination of egg production in a time kinetic (Figure 2A). As previously demonstrated [2], control IL-4Rα^−/lox^ mice behaved as BALB/c mice with peak fecal egg production found at day 7 and subsequently declining thereafter due to a functional host protective immune response [1,3]. Both the IL-4Rα^−/−^ and SM-MHC^Cre^IL-4Rα^−/lox^ mice demonstrated prolonged egg production, with SM-MHC^Cre^IL-4Rα^−/lox^ mice having eggs present in their feces until day 12 postinfection (PI). As expected, IL-4Rα^−/−^ mice demonstrated a chronic infection with eggs present in feces at day 14 PI. Determining the number of worms in the intestine at various time points following infection with N. brasiliensis resulted in comparable worm burdens between IL-4Rα^−/lox^, IL-4Rα^−/−^, and SM-MHC^Cre^IL-4Rα^−/lox^ mice at days 4 and 7 PI. However, at day 10 PI, IL-4Rα^−/lox^ control mice, but not SM-MHC^Cre^IL-4Rα^−/lox^ or IL-4Rα^−/−^ mice, had cleared the worm (Figure 2B), explaining the extended worm fecundity. SM-MHC^Cre^IL-4Rα^−/lox^ mice, but not IL-4Rα^−/−^ mice, showed complete worm expulsion at day 14 PI (Figure 2A). Examination of total serum IgE antibody (Figure 2C) levels showed that SM-MHC^Cre^IL-4Rα^−/lox^ mice responded like the IL-4Rα^−/lox^ mice. Together, these results demonstrate increased susceptibility to N. brasiliensis in smooth muscle cell–specific IL-4Rα–deficient mice with increased parasite burden and delayed worm expulsion.

**Figure 3:**
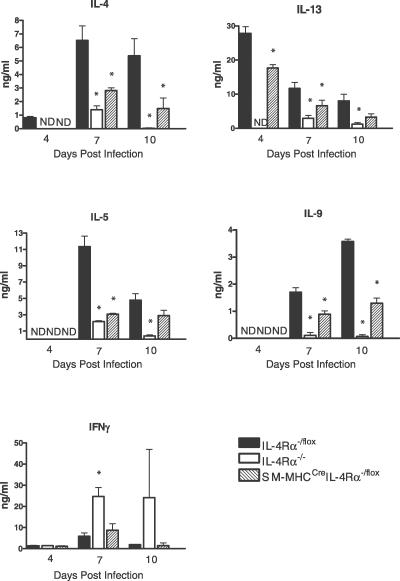
CD4^+^ Lymphocytes Are Essential for N. brasiliensis Clearance MLNs were removed from infected mice on days 4, 7, and 10 PI. CD4^+^ cells were isolated and stimulated with CD3 for 72 h. Supernatants were then analyzed for cytokine production by ELISA as described in Materials and Methods. *Significant difference (*p* < 0.05) from IL-4Rα^−/lox^ mice. Data are representative of four repeated experiments.

**Figure 4:**
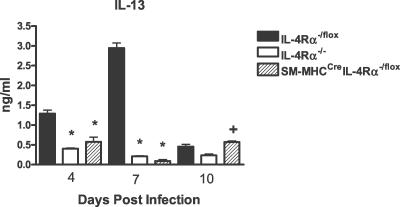
Intestinal IL-13 Levels Are Disrupted in SM-MHC^Cre^IL-4Rα^−/lox^ Mice Intestinal supernatants were analyzed for IL-13 cytokine production by ELISA as described in Materials and Methods. *Significant difference (*p* < 0.05) from IL-4Rα^−/lox^ mice; +significant difference from day 10 PI IL-4Rα^−/−^ mice. Data are representative of three repeated experiments.

T_H_2 cytokines drive protective mechanisms following N. brasiliensis infection [4]. Therefore, cytokine production by anti-CD3–stimulated CD4^+^ T cells purified from mesenteric lymph nodes (MLNs) was analyzed at days 4, 7, and 10 PI. A reduction (*p* < 0.05) of T_H_2 cytokine responses was observed from CD4^+^ T cells of SM-MHC^Cre^IL-4Rα^−/lox^ mice at all time points compared to IL-4Rα^−/lox^ control mice, including IL-4, IL-5, IL-9, and IL-13 (Figure 3). Impairment was comparable to mesenteric CD4^+^ T cell from IL-4Rα^−/−^ mice at day 7 PI. Whereas global IL-4Rα^−/−^ mice shifted to a polarized T_H_1 cytokine response, indicated by the production of interferon γ, this was not observed in infected SM-MHC^Cre^IL-4Rα^−/lox^ mice, which had similar interferon γ levels as IL-4Rα^−/lox^ control mice. In order to ascertain any compensatory cytokine production in the intestine, we examined IL-13 levels from small intestine tissue at days 4, 7, and 10 PI (Figure 4). At days 4 and 7 PI, IL-13 levels were significantly elevated in IL-4Rα^−/lox^ mice compared to IL-4Rα^−/−^ and SM-MHC^Cre^IL-4Rα^−/lox^ mice (*p* < 0.05). By day 10 PI, intestinal IL-13 levels were reduced in IL-4Rα^−/lox^ mice but still significantly higher than those in IL-4Rα^−/−^ mice (*p* < 0.05). SM-MHC^Cre^IL-4Rα^−/lox^ mice, however, also showed significantly higher levels of IL-13 than did IL-4Rα^−/−^ mice at day 10 PI (*p* < 0.05) in accordance with the delayed worm expulsion.

**Figure 5:**
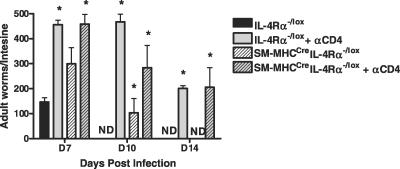
T_H_2 Cytokine Responses Are Impaired in SM-MHC^Cre^IL-4Rα^−/lox^ Mice Mice were injected IP with depleting anti-CD4 antibody 3 d before infection and subsequently every 4 d. Intestinal worm counts were examined at days 7, 10, and 14 PI in IL-4Rα^−/lox^ mice and SM-MHC^Cre^IL-4Rα^−/lox^ mice. *Significant differences compared to IL-4Rα^−/lox^ mice (*p* < 0.05); data are representative of a single experiment.

Reduced T_H_2 responses in the MLNs had no influence on systemic type 2 antibody responses, as there were similar total serum IgG_1_ (unpublished data) and IgE (Figure 2) concentrations in infected SM-MHC^Cre^IL-4Rα^−/lox^ and IL-4Rα^−/lox^ mice. Effective clearance of N. brasiliensis is associated with a CD4-driven T_H_2 cytokine response with IL-13 playing an essential role [1]. In order to confirm a requirement for CD4^+^ T cells in conferring protection, we carried out a CD4^+^ antibody–driven depletion of these cells. Depletion was confirmed using FACS analysis (unpublished data). As expected [15], IL-4Rα^−/lox^–treated mice were unable to clear infection, and CD4^+^ T cells were also essential for clearance in SM-MHC^Cre^IL-4Rα^−/lox^ mice, as depletion resulted in increased adult worm burdens in SM-MHC^Cre^IL-4Rα^−/lox^ mice (Figure 5).

**Figure 6:**
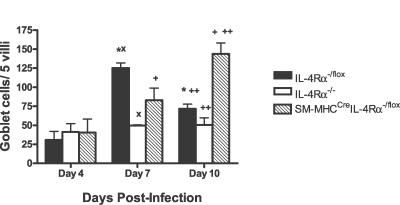
Intestinal Goblet Cell Hyperplasia Is Delayed in SM-MHC^Cre^IL-4Rα^−/lox^ Mice following Infection with N. brasiliensis Mucus-producing goblet cells were visualized using periodic−acid Schiff reagent staining at days 4, 7, and 10 PI. The number of hyperplasic goblet cells per five villi was calculated. Values indicate mean ± SD, with *, x, +, and ++ indicating significant differences between groups (*p* < 0.05). *Significant decrease in hyperplasic goblet cells in IL-4Rα^−/lox^ mice at day 10 PI compared to IL-4Rα^−/lox^ mice at day 7 PI. x, Significantly less hyperplasic goblet cells in IL-4Rα^−/−^ mice than in IL-4Rα^−/lox^ mice at day 7 PI. +, SM-MHC^Cre^IL-4Rα^−/lox^ mice had significantly more hyperplasic goblet cells at day 10 PI than did SM-MHC^Cre^IL-4Rα^−/lox^ mice at day 7 PI. ++, SM-MHC^Cre^IL-4Rα^−/lox^ mice had significantly more hyperplasic goblet cells than did IL-4Rα^−/lox^ mice at day 10 PI. Data are representative of four separate experiments.

T_H_2 cytokine–driven expulsion of N. brasiliensis infections is associated with a concomitant increase in IL-4Rα–dependent intestinal goblet cell hyperplasia and mucus production, a process impaired in IL-4Rα^−/−^ mice [3]. Interestingly, impairment of goblet cell hyperplasia was observed in SM-MHC^Cre^IL-4Rα^−/lox^ mice. At day 7 PI, where SM-MHC^Cre^IL-4Rα^−/lox^ mice showed comparable worm burdens and egg production as Cre-negative IL-4Rα^−/lox^ control mice (Figure 2), qualitative analysis of intestine histology sections, stained with periodic−acid Schiff reagent to visualize goblet cell mucus production, indicated abrogated mucus production in global IL-4Rα^−/−^ mice and a transient reduction of goblet cell hyperplasia in SM-MHC^Cre^IL-4Rα^−/lox^ mice, compared to IL-4Rα^−/lox^ control mice (Figure 5). The mucus production was delayed in SM-MHC^Cre^IL-4Rα^−/lox^ mice as by day 10 PI in goblet cell hyperplasia was comparable to levels observed in IL-4Rα^−/lox^ control mice at day 7 PI (Figure 6).

**Figure 7:**
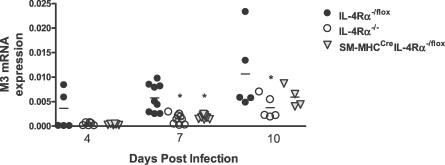
Intestinal Expression of M3 Receptor Is Inhibited by Disrupted Smooth Muscle Cell IL-4Rα Expression mRNA was extracted from intestines of N. brasiliensis infected mice at days 4, 7, and 10 PI. Synthesized cDNA was probed with primers to M3. Increases are normalized against α-actin. Data are representative of two to five experiments per time point. *n* ≥ 4 mice per group. *p < 0.05.

In addition to goblet cell hyperplasia, another proposed mechanism of expulsion of N. brasiliensis from the host is an increased contractile ability of smooth muscle cells [6]. Induction of such contractility is primarily mediated through an acetylcholine-driven cholinergic response mediated by the M3 muscarinic receptor [6,16,17]. We examined mRNA expression levels of the M3 receptor in the intestine of mice at days 4, 7, and 10 PI (Figure 7). At day 4 PI, no significant difference was noted between groups, although a trend for higher expression in IL-4Rα^−/lox^ mice was noted. We found that at peak infection (day 7 PI), IL-4Rα^−/lox^ mice had significantly higher (*p* < 0.05) expression levels of M3 than both IL-4Rα^−/−^ and SM-MHC^Cre^IL-4Rα^−/lox^ mice. By day 10 PI, IL-4Rα^−/−^ mice still showed a significantly lower level of M3 mRNA expression compared to IL-4Rα^−/lox^ mice. However, SM-MHC^Cre^IL-4Rα^−/lox^ mice showed increased M3 expression compared to that on day 7 PI. This important result is the first report of IL-4Rα expression having an effect on the expression of acetylcholine receptors in vivo.

Together, these results show smooth muscle IL-4Rα plays an important role in the regulation of both draining lymph and intestinal cytokine production, goblet cell hyperplasia, and acetylcholine responsiveness. Disruption of these responses in the SM-MHC^Cre^IL-4Rα^−/lox^ mice results in delayed expulsion of the parasites.

## Discussion

This work provides the first description of the generation, characterization, and functional analysis of a smooth muscle cell–specific IL-4Rα–deficient mouse model. Disruption of IL-4Rα expression in smooth muscle cells was applied to a disease model where smooth muscle cells are proposed to play an important role in the resolution of infection, namely, a gastrointestinal nematode infection [6].

Clearance of nematode pathogens from the intestine is considered to require a number of physiological and immunological responses by the host. Increased intestinal contractions [6], increased mucus production [18], and elevated levels of T_H_2-associated antibodies and cytokines [3] are all mechanisms induced by nematode infection. Wild-type mice infected with N. brasiliensis cleared the infection at day 9 PI, while SM-MHC^Cre^IL-4Rα^−/lox^ mice had an impaired ability to clear the nematode until day 12 PI. We demonstrated this impairment to be associated with a delay in goblet cell hyperplasia and the subsequent influx of mucus into the lumen of the host intestine. These physiological disruptions were related to an inability of the host to amplify appropriate cytokine production both locally and by CD4^+^ T cells from the draining MLNs.

A number of authors have demonstrated nematode-induced amplification of intestinal smooth muscle contractions to be dependent on IL-13/IL-4Rα/STAT-6 signaling. Isolated strips of smooth muscle from the small intestine of N. brasiliensis–infected STAT6^−/−^ mice have a significantly decreased tensile potential in vitro [6]. Depressed contractile ability was also observed in other nematode models in the absence of STAT-6 [6,9]. The significance of these nematode-induced contractions in the resolution of infection remains unclear. Recent work has demonstrated that the serotonin receptor 5-HT_2a_ is a potent inducer of IL-13– and N. brasiliensis–dependent intestinal contractions. However, specific inhibition of 5-HT_2a_ failed to affect the ability of the host to resolve infection [19]. We demonstrate a striking reduction in the expression of the acetylcholine M3 receptor in SM-MHC^Cre^IL-4Rα^−/lox^ and IL-4Rα^−/−^ mice following N. brasiliensis infection. The M3 expression data we present here are similar to those of 5-HT_2a_ in response to N. brasiliensis infection. However, the potential role of M3 in mediating expulsion of intestinal parasites is more compelling. M3^−/−^ mice are incapable of eliciting smooth muscle contractions [16]; this is not the case in 5-HT_2a_^−/−^ mice [20]. Previous studies have demonstrated IL-13– and STAT-6–dependent increases in acetylcholine-induced smooth muscle contractions in tissue from N. brasiliensis–infected mice [6]. M3 is the principal acetylcholine receptor in smooth muscle and drives 75% of the contractile response in the small intestine [16]. As such, our demonstration of significant inhibition of M3 expression in IL-4Rα–deficient mice is compelling in vivo evidence of IL-4Rα–muscarinic receptor interactions contributing to proposed muscle hypercontractility–aided nematode expulsion [8].

In addition to contractile responses, host epithelial responses constitute a second major physiological response to the parasite. This response varies according to parasite; in the case of the intraepithelial nematode *Trichuris muris,* expulsion is driven by epithelial cell turnover [21]. The principal aspect of this response to the luminal dwelling N. brasiliensis is induction of goblet cell–driven mucus production. Goblet cell–derived mucus is essential for clearance of N. brasiliensis infection [22,23]. Secreted mucus directly affects viability of the worms through inhibition of parasite motility [24,25] and ability to feed [26]. Pathogen-induced mucus production is strongly influenced by the host immune response. A deficiency in T_H_2 polarization severely impairs the ability of goblet cells to secrete mucus and expel N. brasiliensis [18]. Mucus production is also modulated by the enteric nervous system via innervation of mucosal mast cells [27] and goblet cells [28]. Innervation of epithelial mucus-producing cells is also important for the host mucosal response to N. brasiliensis infection [29,30]. Previous studies have established the importance of this epithelial response, the most significant cells for effective expulsion being the mucus-producing goblet cells. This body of work combined with the data we present suggests that smooth muscle cells may represent an intermediate zone of signal transduction between the epithelium and MLNs. Disruption of the ability of the smooth muscle cells to respond to IL-4Rα ultimately results in a delayed mucosal response and depressed MLN cytokine production.

Prolonged N. brasiliensis infection, due to a deficiency in smooth muscle cell expression of IL-4Rα, may therefore be a result of the host's inability to mount an effective mucus response. Delayed mucus responses to N. brasiliensis infection are associated with an impaired T_H_2 response [18]. The delayed expulsion we report here is then explained by the reduced MLN CD4^+^ T_H_2 response (Figure 3), delaying mucus production (Figure 6) through inhibition of smooth muscle responsiveness to neurotransmitters (Figure 7) and cytokines. The depressed T_H_2 response we suggest to be a result of smooth muscle cells being unable to react effectively to the key smooth muscle contraction amplifying cytokine IL-13 and the neurotransmitter acetylcholine [6] sufficiently to stimulate rapid cytokine production in the MLNs. Parasite clearance would then be more reliant on local effector lymphoid tissue responses [31]. The resulting recovery in response to infection and its eventual clearance in the SM-MHC^Cre^IL-4Rα^−/lox^ mouse may then be explained by local responses in the intestine providing a sufficient, albeit delayed and reduced compensatory response which induces the eventual disease-resolving response (Figure 4).

**Figure 8:**
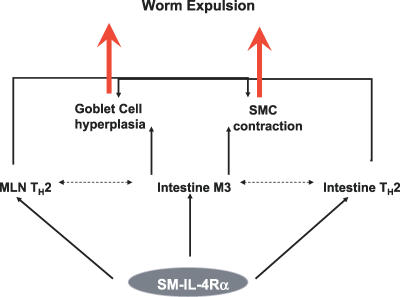
Role of Smooth Muscle IL-4Rα in N. brasiliensis Infection Solid arrows represent demonstrated effects of smooth muscle IL-4Rα on the host response to N. brasiliensis infection. Dotted arrows indicate other potential and/or likely interactions.

In conclusion, we have demonstrated in vivo a significant role for smooth muscle cell IL-4Rα in the optimal resolution of a gastrointestinal nematode infection. Deletion of smooth muscle IL-4Rα significantly disrupts the host ability to resolve infection with N. brasiliensis. We demonstrate severe disruption of both known and proposed mediators of expulsion. Depressed M3 receptor expression, delayed goblet cell hyperplasia, disruption of CD4^+^ MLNs, and intestinal cytokine production provide compelling evidence for an important role in the induction of both physiological effector mechanisms and immunological mediators of expulsion. Together, these data are suggestive of smooth muscle IL-4Rα being an important inducer of T_H_2 cytokine signaling from the lymph node and tissue and goblet cell hyperplasia and having a striking effect on the key smooth muscle contraction–inducing M3 muscarinic receptor (Figure 8).

## Materials and Methods

### Generation and genotyping of conditional IL-4Rα–deficient mice.

SM-MHC^Cre^ mice were a kind gift from Gary K. Owens, Charlottesville, Virginia, United States [14,32]. SM-MHC^Cre^ mice were backcrossed to Balb/c for nine generations and then intercrossed with IL-4Rα^−/−^ mice (*n* = 30). These mice were then mated with IL-4Rα^−/lox^ mice (*n* = 2) to generate SM-MHC^Cre^IL-4Rα^−/lox^ mice. Transgene negative littermates (IL-4Rα^−/lox^) were used as controls in all experiments. Mice were housed under specific pathogen-free barrier conditions in the University of Cape Town animal facility. All work was approved by the University of Cape Town animal ethics board.

### Genotyping.

Specific PCR primer pairs were for the IL-4Rα, 5′-GTACAGCGCACATTGTTTTT-3′ and 5′-CTCGGCGCACTGACCCATCT-3′; deletion, 5′-GGCTGCCCTGGAATAACC-3′ and 5′-CCTTTGAGAACTGCGGGCT-3′; LoxP, 5′-CCCTTCCTGGCCCTGAATTT-3′ and 5′-GTTTCCTCCTACCGCTGATT-3′; and Cre, 5′-ATGCCCAAGAAGAAGAGGAAGGT-3′ and 5′-GAAATCAGTGCGTTCGAACGCTAGA-3′. PCR conditions were as follows: 94 °C for 1 min, 94 °C for 30 s, 57 °C for 30 s, and 72 °C for 1 min for 40 cycles on an MJ thermocycler (Biozym Diagnostik, http://www.biozym.com).

### Analysis of IL-4Rα expression by FACS.

A single cell suspension of smooth muscle cells was prepared as previously described [33] along with lymph node cells. For the intracellular stain, cells in single cell suspension were incubated with 1% normal rat serum and stained with rat anti-mouse IL-4Rα–PE (mIL-4RM-1; BD Biosciences, http://www.bdbiosciences.com). Stained cells were then washed, fixed in 2% paraformaldehyde, permeabolized with saponin, preblocked with 2% NRS and Fc block (2.4G2), and stained with anti–α-actin FITC (Abcam, http://www.abcam.com) or isotype control IgG2a-FITC (BD Biosciences). For lymphocyte staining, anti–CD3-FITC, anti–CD19-PE, and anti–IL-4Rα biotin in combination with SA-APC were used to identify lymphocyte subsets (BD PharMingen, http://www.bdbiosciences.com). Nonviable cells were stained with 7-AAD and excluded from analysis (Sigma, http://www.sigmaaldrich.com). Acquisition was performed using FACSCalibur and cells analyzed using Cellquest (Becton Dickinson, http://www.bd.com).

### Infection studies.

Mice were injected subcutaneously with 750 N. brasiliensis L3 larva (kindly provided by Klaus Erb, Wurzburg, Germany). Analysis of parasite eggs in feces was carried out using the modified McMaster technique [34]. Adult worm burdens were determined as previously described [3].

### CD4^+^ T-cell depletion.

CD4^+^ T cells were depleted from mice by intraperitoneal injection of 0.5 mg of anti-CD4^+^ monoclonal antibody (clone GK1.5) 3 d prior to infection. Mice received booster injections every 3 d to maintain depletion. Effective depletion was confirmed by FACS analysis.

### Ex vivo restimulation of lymphocytes.

CD4^+^ T cells were purified from pooled MLNs at days 7 and 10 PI. Enrichment was carried with a negative selection. Briefly, cells in single cell suspension were stained with anti-CD8, CD11b, GR-1, B220, and CD16/32. Stained cells were depleted using goat anti-rat IgG–coated magnetic beads (Biomag Beads; Qiagen, http://www.qiagen.com). Cell purity was approximately greater than 98%. CD4^+^ T cells were restimulated for 72 h with anti-CD3 (clone 145–2C11; 20 μg/ml). Supernatants were then collected and stored at −80 °C until analysis.

### ELISA analysis.

Cytokines in supernatants and serum antibody isotype levels from infected animals were determined as previously described [35].

### Histology.

Tissue samples were fixed in a neutral buffered formalin solution. Following embedding in paraffin, samples were cut into 5-μm sections. Sections were stained with hematoxylin and eosin or periodic−acid Schiff reagent. The number of positively stained cells per five villi were counted by light microscopy. All samples were randomized and counted in a blinded fashion.

### RT-PCR.

RNA was extracted from the intestine of infected mice with the use of Tri-reagent (Sigma), and cDNA was synthesized using the ImProm-II Reverse Transcription System (Promega, http://www.promega.com). M3 cDNA was amplified using the following primers: 5′-CGG AAA AGG ATG TCG-3′ and 5′-GGC ACT CGC TTG TGA A-3′. Data were normalized using the α-actin housekeeping gene.

### Statistics.

Values are given as mean ± SD, and significant differences were determined using the Mann-Whitney *U* test.

## Supporting Information

### Accession Numbers

The GenBank (http://www.ncbi.nlm.nih.gov/Genbank) accession numbers for the genes and gene products discussed in this paper are IL-13 (16163), IL-4Rα (16190), STAT-6 (20852), M3 (12671), smooth muscle myosin heavy chain (17880), and α-actin (11475).

## Abbreviations

FACS: fluorescence-activated cell sorting analysis
GMF: geometric mean fluorescence
IL-4R: interleukin 4 receptor
MLN: mesenteric lymph node
PI: postinfection
SM-MHC: smooth muscle myosin heavy chain
STAT-6: signal transducer and activator of transcription 6
T helper 2: T_H_2
