# Correction: Delayed Goblet Cell Hyperplasia, Acetylcholine Receptor Expression, and Worm Expulsion in SMC-Specific IL-4Rα–Deficient Mice

**DOI:** 10.1371/journal.ppat.0030037

**Published:** 2007-04-27

**Authors:** William G. C Horsnell, Antony J Cutler, J. Claire Hoving, Helen Mearns, Elmarie Myburgh, Berenice Arendse, Fred D Finkelman, Gary K Owens, Dave Erle, Frank Brombacher

In *PLoS Pathogens*, volume 3, issue 1: doi: 10.1371/journal.ppat.0030001

The third author's name was incorrectly listed as Claire J. Hoving. The correct name is J. Claire Hoving.

